# HHO5 balances the plant nitrogen diet

**DOI:** 10.1093/plcell/koag241

**Published:** 2026-08-07

**Authors:** Luciano M Di Fino

**Affiliations:** Assistant Features Editor, The Plant Cell, American Society of Plant Biologists; Umeå Plant Science Centre (UPSC), Department of Forest Genetics and Plant Physiology, Swedish University of Agricultural Sciences, Umeå 901 83, Sweden

Nitrogen fuels plant growth, shaping root architecture, photosynthesis, biomass accumulation, and grain yield. Fertilization has helped sustain crop productivity, but plants recover only a fraction of the nitrogen applied to soils. This inefficiency carries economic and environmental costs and has made improved nitrogen-use efficiency a major goal in agriculture (Raun and Johnson 1999; Li et al. 2017). Achieving this goal requires a clearer understanding of how plants sense nitrogen availability and adjust uptake, metabolism, and growth to match their nutritional status.

In nature, plants encounter 2 major types of nitrogen sources in the soil. Inorganic sources, such as nitrate and ammonium, provide major routes for nitrogen acquisition. By contrast, organic compounds such as glutamate and glutamine can also contribute to nitrogen nutrition and transport. These nitrogen species function not only as nutrients but also as signals that trigger distinct transcriptional and physiological responses. Nitrate perception involves regulators such as the NRT1.1 transceptor and the nitrate-binding transcription factor NLP7 (Bouguyon et al. 2015; Liu et al. 2022), While these components have provided important insights into nitrate sensing and signaling, how plants translate nitrogen dose and nitrogen species into coordinated cellular and physiological responses remains less well understood. Recent transcriptomic studies identified transcription factors that respond within minutes to nitrogen treatments, including members of the HRS1-HOMOLOG (HHO) family. Building on these observations, **Hinckley and colleagues (Hinckley et al. 2026)** focused on HHO5, an early nitrogen-responsive transcription factor, to ask how plants balance inorganic nitrate signals with organic nitrogen status to coordinate nitrogen uptake, signaling, and growth.

The authors first established that Arabidopsis roots respond quantitatively to nitrogen supply. Increasing nitrogen doses reprogrammed the root transcriptome, activating genes involved in nitrate assimilation, glutamate metabolism, cytokinin signaling, and other nitrogen-related pathways. HHO5 stood out among nitrogen-responsive transcription factors because it is phylogenetically distinct from other HHO transcription factors, responds to nitrogen dose, and shows preferential expression in the phloem, a cell-type that transports nitrogen throughout the plant. These features suggested that HHO5 could act as a regulatory hub linking local nitrogen perception with systemic nitrogen responses. The authors then compared wild-type plants with *hho5* mutants and found that HHO5 controls the amplitude of nitrogen-dose responsive gene expression. These transcriptional changes had developmental consequences: *hho5* mutants showed reduced growth responses to increasing nitrogen.

At the molecular level, the authors found that HHO5 directly represses nitrate-response genes, including those associated with inorganic nitrogen uptake. By contrast, HHO5 promotes expression of genes linked to organic nitrogen responses and stress-related pathways. This dual activity raised a key mechanistic question: how can HHO5 repress some targets while activating others? The authors identified the transcription factor WRKY21 and showed that it enhances HHO5-dependent gene induction. This supports a model in which HHO5 represses some nitrate-responsive targets directly, while activating other organic N-responsive genes indirectly through cooperation with partner transcription factors. By integrating these direct and indirect targets into a gene regulatory network, the authors showed that HHO5 controls a substantial fraction of the nitrogen-dose response and connects inorganic nitrate signaling with organic nitrogen pathways.

Hinckley et al. add another important dimension by showing that HHO5 links nitrogen dose perception with the balance between inorganic nitrate uptake and organic nitrogen status. The T-DNA *hho5* mutant took up more inorganic nitrogen under low-nitrogen conditions and showed altered root responses to higher doses of organic-N (glutamate). These N dose–dependent phenotypes support the idea that HHO5 limits nitrate uptake when organic nitrogen signals indicate nitrogen sufficiency, while promoting responses to organic nitrogen. Together, the results reveal HHO5 as a dose-dependent regulatory switch that coordinates nitrogen acquisition, internal nitrogen status, root growth, and plant development (Fig. 1). By linking nitrate uptake to organic nitrogen signaling, HHO5 may help plants conserve energy and fine-tune nitrogen use across fluctuating soil environments.

**Figure 1:**
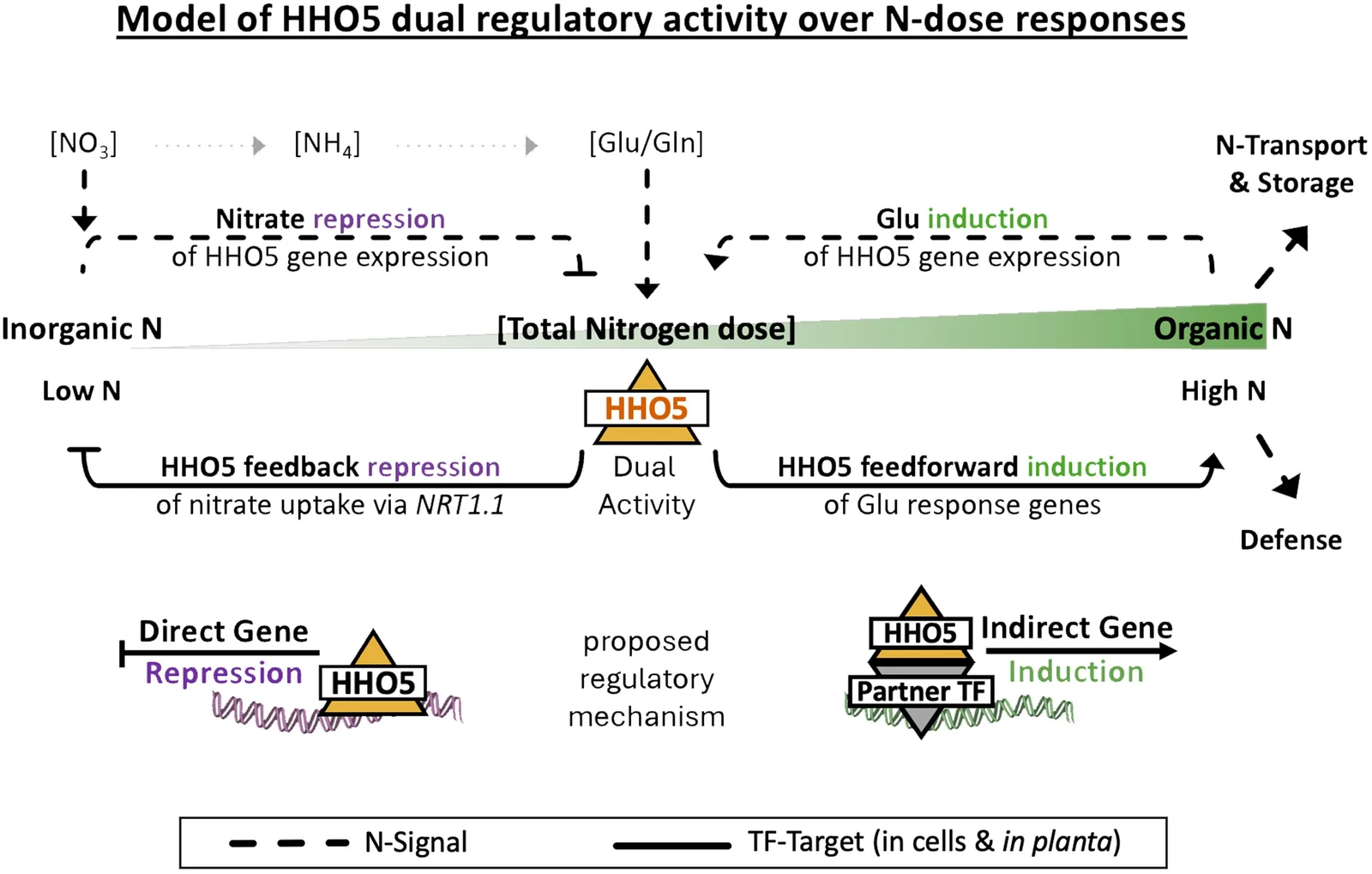
Proposed model of HHO5 dual regulatory activity over nitrogen-dose responses. HHO5 acts as a nitrogen-responsive regulator that represses nitrate uptake under low inorganic nitrogen while promoting glutamate-responsive gene expression under higher organic nitrogen conditions. Adapted from Hinckley et al., grapshical abstract.

This work represents an important advance in understanding how plants coordinate inorganic and organic nitrogen signaling with physiological responses. Specifically, nitrate represses HHO5 expression, whereas organic nitrogen signals, such as glutamate, induce it. Still, several questions remain. One promising future direction would be to explore how the nitrogen-regulated HHO5 mechanism intersects with plant defense. By directly activating glutamate receptor genes (GLRs), HHO5 may help sense defense-related signals and suppress nitrate uptake to conserve energy during pathogen attack.

## Recent related articles in *The Plant Cell*

- **Xuelian Zheng et al. 2025** discovered that SnRK1 α-catalytic subunit (SnRK1α1) in tomato enhanced nitrate uptake capacity and seedling tolerance through apoplastic ROS signaling.
- **Chao Bian et al. 2025** showed that nitrate-responsive gene circuits in plants share some conserved regulators between Arabidopsis and tomato, especially NLP and ARF transcription factors.

## Data Availability

No new data were generated or analysed in support of this research.
